# Integrated Metabolomic and Proteomic Analyses of Adventitious Rooting in *Cucumis melo* Under Waterlogging Stress

**DOI:** 10.3390/biology15141185

**Published:** 2026-07-17

**Authors:** Huanxin Zhang, Qian Chen, Guoquan Li, Huifang Lv, Lihong Guo, Chenghe Ma, Xinlong Hu

**Affiliations:** 1 Nanchang Key Laboratory of Germplasm Innovation and Utilization of Fruit and Tea, Institute of Horticulture, Jiangxi Academy of Agricultural Sciences, Nanchang 330200, China; zhanghuanxin150@163.com (H.Z.);; 2 School of Biology and Food Engineering, Hefei Normal University, Hefei 230601, China; 3 Jiangxi Agricultural Technology Extension Center, Nanchang 330046, China

**Keywords:** adventitious root, *Cucumis melo*, metabolome, proteome, waterlogging stress

## Abstract

Waterlogging-induced hypoxic stress severely impairs vegetative growth and crop yield of melon (*Cucumis melo* L.). The formation of adventitious roots represents a critical morphological adaptive strategy for melon seedlings to alleviate hypoxic damage and maintain viability under waterlogging conditions. Nevertheless, the synergistic molecular regulatory mechanisms governing waterlogging-triggered adventitious root development in melon remain largely uncharacterized at the proteomic and metabolomic layers. In this study, the waterlogging-tolerant melon line ‘L8’ with superior adventitious root production capacity was exposed to waterlogging treatment, and hypocotyl tissues were harvested at 0, 24, 48 and 72 h post-waterlogging for untargeted metabolomic and proteomic analyses. A total of 1337 differentially accumulated metabolites (DAMs) and 2898 differentially expressed proteins (DEPs) were identified across the pairwise comparisons. Functional enrichment analyses of DAMs and DEPs indicated that pathways related to linoleic acid metabolism, α-linolenic acid metabolism, phenylpropanoid biosynthesis, biosynthesis of secondary metabolites, and glutathione metabolism were centrally implicated in adventitious rooting induced by waterlogging. At the protein level, pivotal functional proteins associated with anaerobic respiration (pyruvate decarboxylase, alcohol dehydrogenase), ethylene biosynthesis (1-aminocyclopropane-1-carboxylate oxidase), cell wall remodeling and antioxidant defense were significantly up-regulated throughout adventitious root development. In addition, three transcription factors, namely the GRAS family protein MELO3C025904.1, MYB-related protein MELO3C007640.1, and ZF-HD protein MELO3C022921.1, exhibited differential expression across different time points compared to the control. Moreover, metabolomic profiling identified three prominent metabolites with regulatory functions, encompassing the terpenoid acorusnol, the piperidine alkaloid 1,4′-bipiperidine-1′-carboxylic acid, and the flavonoid 4′,7-dihydroxy-2′-methoxy-3′-prenylisoflavan. Omics correlation analysis revealed extensive concordance between metabolomic and proteomic profiles. Nine core DAMs, including N-methylserotonin, Val-Val and glyuranolide, were tightly correlated with hundreds of DEPs and key transcription factors, constructing a complex regulatory network governing waterlogging stress acclimation and adventitious root morphogenesis. This study systematically characterizes the coordinated proteomic and metabolomic reprogramming underlying waterlogging-induced adventitious root formation in melon. These findings deepen our understanding of the molecular mechanism of waterlogging tolerance and provide valuable candidate genes and metabolic targets for genetic improvement of waterlogging resistance in melon.

## Simple Summary

Waterlogging stress severely suppresses the growth and survival of melon plants primarily by inducing hypoxia in submerged tissues. To survive hypoxic conditions caused by waterlogging, the hypocotyls of melon seedlings develop abundant adventitious roots. In the present study, integrated metabolomic and proteomic analyses were performed to systematically investigate waterlogging-induced adventitious root formation in melon. The results revealed that critical functional proteins related to anaerobic respiration, ethylene biosynthesis, cell wall remodeling and antioxidant defense, as well as a series of transcription factors from GRAS, MYB-related and ZF-HD families, were markedly regulated during adventitious rooting. Moreover, acorusnol, classified as a terpenoid, 1,4′-bipiperidine-1′-carboxylic acid, categorized as a piperidine alkaloid, and 4′,7-dihydroxy-2′-methoxy-3′-prenylisoflavan, annotated as a flavonoid, displayed vital roles in waterlogging response. Multi-omics integration analysis further demonstrated that core metabolites and regulatory proteins constituted a sophisticated molecular network that orchestrates waterlogging adaptation and adventitious root development. Our findings elucidate the proteomic and metabolomic molecular mechanisms underlying melon waterlogging tolerance, providing valuable genetic and metabolic resources for the breeding of waterlogging-tolerant melon cultivars.

## 1. Introduction

Water is one of the core factors for crop survival, but excessive water, waterlogging, threatens the normal growth and development of crops throughout the lifecycle. In recent years, the combined effects of global warming, ecological degradation, and the increasing frequency of extreme weather events, in conjunction with short-term extreme precipitation, heavy soil texture and poor soil drainage, have led to the frequent occurrence of waterlogging stress [1,2]. It is estimated that waterlogging stress affects approximately 16% of the world’s agricultural production areas, thereby emerging as a key natural disaster constraining the high-quality development of agriculture and the enhancement of crop productivity [3,4]. China is particularly vulnerable to waterlogging; notably, in the middle and lower reaches of the Yangtze River Basin, persistent waterlogging conditions prevail due to high rainfall, elevated groundwater tables, and inadequate drainage systems [2]. Under waterlogging stress, soil pores are filled with water, which blocks air diffusion into the soil and consequently induces hypoxia in crop roots. This hypoxic condition forces a metabolic shift in root respiration from the aerobic to the anaerobic pathway, triggering the excessive accumulation of toxic metabolites such as ethanol, acetaldehyde, and lactic acid. The accumulation of these toxic substances subsequently leads to root rot and necrosis, impairing the root functions of water uptake and mineral nutrient absorption. Such root dysfunction further elicits a cascade of aboveground physiological disorders, including stomatal closure, chlorophyll degradation, and a drastic reduction in photosynthetic efficiency. The consequent severe shortage in the synthesis and supply of usable sugars ultimately compromise the normal growth, development, and yield potential of crops [5,6].

To cope with waterlogging stress, different crop species have evolved specific morphological adaptive mechanisms, including leaf epinasty, internode elongation, aerenchyma formation and adventitious root development. Among these, adventitious root formation is a crucial and widespread morphological response pathway adopted by numerous crops to adapt to waterlogging stress [7]. It has been reported that crops such as wheat [8], maize [9], tomato [10], bitter melon [11], cucumber [12], sponge gourd [13] and melon [14] can alleviate waterlogging-induced plant damage via the formation of adventitious roots. These adventitious roots feature a rapid growth rate, short generation cycle, well-developed cortex and high propensity for aerenchyma formation. Under waterlogging stress, they can rapidly replace the decayed original root system, restore the uptake and translocation of water and mineral nutrients in plants, and thus ensure the normal progression of photosynthetic and metabolic processes in the aboveground tissues [15]. Meanwhile, the well-developed aerenchyma can efficiently mediate oxygen transport to underground tissues, effectively alleviate hypoxic stress in the original root system and reduce the accumulation of toxic substances produced by anaerobic respiration [8,16]. Collectively, these traits of adventitious roots confer improved waterlogging tolerance in crops.

Melon (*Cucumis melo* L.) is an economically important cucurbit crop widely cultivated worldwide, yet its production is frequently constrained by waterlogging stress. The formation of adventitious roots on the hypocotyl is a critical morphological adaptation that allows melon seedlings to endure such stress and maintain essential life processes [7,14]. Although our previous transcriptomic analysis provided preliminary insights into the regulatory networks governing adventitious root formation under waterlogging conditions [7], transcriptomic data alone are insufficient to capture the actual execution of biological processes. As the primary direct executors and final functional effectors of gene action, proteins and metabolites are hypothesized to play indispensable roles in regulating waterlogging-triggered adventitious rooting. We further propose that these molecules interact to form a sophisticated and coordinated network that orchestrates adaptive responses to waterlogging and drives developmental reprogramming for adventitious root organogenesis. To systematically verify this hypothesis, the present study integrated proteomic and metabolomic approaches to dissect dynamic molecular changes in melon hypocotyls at 0, 24, 48 and 72 h post-waterlogging treatment. This integrated multi-omics analysis aims to identify key proteins and metabolic pathways involved in adventitious rooting induced by waterlogging. The findings will deepen our understanding of the molecular basis of waterlogging tolerance in melon and provide a theoretical foundation for the genetic improvement of stress resistance in this crop.

## 2. Materials and Methods

### 2.1. Plant Material and Stress Treatment

The experimental material used in this study was the waterlogging-tolerant melon line ‘L8’, cultivated by the Institute of Horticulture at the Jiangxi Academy of Agricultural Sciences. This material is notable for its high propensity for prolific adventitious root formation in response to waterlogging stress [7]. Uniformly plump seeds were soaked in distilled water for 4 h, thoroughly rinsed to remove surface mucilage, and then incubated for germination at a constant temperature of 28 °C. Germinated seeds were sown in plastic pots (7 cm × 7 cm × 7 cm) filled with a composite growth substrate composed of peat, vermiculite, and perlite in a volumetric ratio of 3:1:1. Seedlings were cultivated in a controlled growth chamber with a 12 h light/12 h dark photoperiod, day/night temperatures of 28 °C/18 °C, a photosynthetic photon flux density of 300 μmol·m^−2^·s^−1^, and relative humidity maintained at 70–80%. After four weeks, healthy and uniform seedlings were subjected to waterlogging stress treatment in plastic containers. The water level was strictly maintained at the top of the hypocotyls, with water added daily to preserve a consistent depth. The hypocotyls below the water surface were harvested at 0, 24, 48, and 72 h after the initiation of waterlogging stress, with the 0 h samples serving as the control group. Samples for proteomic profiling were designated as P0h, P24h, P48h, and P72h, while those for metabolomic analysis were labeled as M0h, M24h, M48h, and M72h, respectively. There were three biological replicates for proteomics and six replicates for metabolomics, with 30 seedlings utilized for each replicate. Immediately after collection, all samples were flash-frozen in liquid nitrogen and stored at −80 °C until subsequent proteomic and metabolomic analyses. For triphenyltetrazolium chloride (TTC) staining, hypocotyl samples collected at 0, 24, 48, 72 and 96 h post-waterlogging were incubated in 0.4% TTC staining solution for 2 h and then photographed.

### 2.2. Untargeted Metabolomic Analysis

For untargeted metabolomic analysis, 100 mg of hypocotyl samples were prepared from each treatment and subjected to subsequent procedures. The metabolite extraction method was consistent with that described in our previously published article [14]. Following ultrasonic extraction at low temperature and subsequent centrifugation, the supernatant was collected for LC-MS/MS analysis. To ensure system conditioning and quality assurance, a pooled quality control (QC) sample was created by mixing equal volumes of all individual samples. The resulting QC samples were processed and analyzed in strict accordance with the same analytical protocol applied to the test samples. LC-MS/MS analysis of all samples was conducted using a UHPLC-Q Exactive system at Majorbio Bio-Pharm Technology Co., Ltd. (Shanghai, China). In the present study, a BEH C18 column (100 mm × 2.1 mm, 1.7 µm) and a BEH amide column (100 mm × 2.1 mm, 1.7 µm), both from Waters (Milford, MA, USA), were employed for chromatographic separation. For the BEH C18 column, the mobile phases consisted of 2% acetonitrile in water (containing 0.1% formic acid) as solvent A and 0.1% formic acid in acetonitrile as solvent B. For the BEH amide column, the mobile phases consisted of 95% acetonitrile in water (containing 5 mM ammonium acetate and 0.04% acetic acid) as solvent A and 5% acetonitrile in water (containing 10 mM ammonium acetate and 0.04% acetic acid) as solvent B. The injection volume was 3 μL, the flow rate was 0.40 mL/min, and the column temperature was maintained at 40 °C. Following electrospray ionization, mass spectrometric signals were acquired in both positive and negative ion scanning modes.

Raw LC/MS data preprocessing was performed using Progenesis QI software (Version 3.0) (Waters Corporation, Milford, MA, USA). The data matrices obtained from the database searches of the two columns were combined, deduplicated, and subsequently uploaded to the Majorbio Cloud Platform (https://cloud.majorbio.com, accessed on 29 November 2025) for further analysis. The data processing method was consistent with that described previously [14]. Metabolites with a variable importance in projection (VIP) value > 1 and a *p*-value < 0.05, derived from the OPLS-DA model and Student’s t-test, respectively, were identified as differentially accumulated metabolites (DAMs). The DAMs were mapped to their corresponding biochemical pathways through metabolic enrichment and pathway analysis based on the KEGG database (http://www.genome.jp/kegg/, accessed on 29 November 2025). Additionally, these DAMs were further categorized according to the metabolic pathways they participate in and the biological functions they exert. The Python package “scipy.stats” (Version 1.0.0) (https://docs.scipy.org/doc/scipy/, accessed on 29 November 2025) was utilized to perform enrichment analysis to identify the most relevant biological pathways for the treatments.

### 2.3. Proteomic Analysis

Protein extraction was performed using the BPP method [17], and protein concentration was determined by the BCA assay. Subsequently, 10 µg of protein from each sample was loaded for SDS-PAGE electrophoresis. For protein digestion, 100 μg of protein solution was aliquoted from each sample, and triethylammonium bicarbonate (TEAB) buffer was added to achieve a final concentration of 100 mM. Tris(2-carboxyethyl)phosphine (TCEP) was then added to a final concentration of 10 mM, followed by incubation at 37 °C for 60 min. Iodoacetamide was added to a final concentration of 40 mM, and the reaction was conducted in the dark at room temperature for 40 min. After centrifugation at 10,000× *g* for 20 min, the pellet was collected and fully dissolved in 100 µL of 100 mM TEAB. Trypsin was subsequently added and incubated at 37 °C overnight. After trypsin digestion, the peptides were dried using a vacuum pump, re-solubilized with 0.1% trifluoroacetic acid (TFA), desalted, and dried again. Finally, the peptides were quantified using ultraviolet spectrophotometry. The peptides were analyzed using a VanquishNeo (Thermo, Waltham, MA, USA) coupled with a Compass HyStar (Bruker, Germany) at Majorbio Bio-Pharm Technology Co., Ltd. (Shanghai, China). Solvent A consisted of water with 2% acetonitrile and 0.1% formic acid, while solvent B comprised water with 80% acetonitrile and 0.1% formic acid. The chromatography run time was set to 8 min. Data-independent acquisition (DIA) data were acquired using a timsTOF HT mass spectrometer (Bruker, Germany) operated in DIA mode. Positive ion mode was used for detection; the ion source voltage was set to 1.5 kV, and the mass spectrometry scan range was 100–1700 *m*/*z*.

Raw DIA data were imported into the Spectronaut software (Version 19) for database searching (http://cucurbitgenomics.org/v2/organism/23, accessed on 26 November 2025). The parameter settings were as follows: peptide length ranged from 7 to 52 amino acids; the enzyme cutting site was trypsin/P; the maximum allowed missed cleavage sites were 2; carbamidomethylation of cysteines was designated as fixed modification, while oxidation of methionines and protein N-terminal acetylation were classified as variable modifications; the protein false discovery rate (FDR) was set at ≤0.01; the peptide FDR was also set at ≤0.01; peptide confidence was required to be ≥99%; and the extracted ion chromatogram (XIC) width was limited to ≤75 ppm. The protein quantification method employed was MaxLFQ. All data were uploaded to the Majorbio Cloud Platform (https://cloud.majorbio.com) for subsequent bioinformatic analysis. Differentially expressed proteins (DEPs) were defined as those exhibiting a fold change (FC) > 1.5 or <0.67, along with a *p*-value < 0.05. Functional classification and metabolic pathway enrichment analysis of the identified DEPs were conducted using the Gene Ontology (GO) database (http://geneontology.org/, accessed on 29 November 2025) and the Kyoto Encyclopedia of Genes and Genomes (KEGG) database (http://www.genome.jp/kegg//, accessed on 26 November 2025).

### 2.4. Combined Metabolomic and Proteomic Analysis

An integrated analysis of metabolomic and proteomic datasets was performed via sparse generalized canonical correlation discriminant analysis. This algorithm was implemented in the Data Integration Analysis for Biomarker Discovery using Latent Components (DIABLO) module of the R package mixOmics (Version 1.0.6). Prior to DIABLO-based integration, both normalized metabolomic and proteomic datasets were subjected to logarithmic transformation [18]. The two-way orthogonal partial least squares (O2PLS) analysis was carried out via the R package OmicsPLS (Version 2.0.2). Additionally, the correlation between differentially accumulated metabolites and differentially expressed proteins was quantified using Python (Version 2.7.10).

### 2.5. Quantitative Reverse Transcription-PCR (qRT-PCR) Verification

Total RNA was isolated from P0h, P24h, P48h, and P72h samples. RNA extraction was conducted with the RNAprep Pure Plant Kit (Tiangen Biotech, Beijing, China) in strict accordance with the manufacturer’s instructions. First-strand cDNA was synthesized using the TransScript^®^ All-in-One First-Strand cDNA Synthesis SuperMix (TransGen Biotech, Beijing, China). The qRT-PCR was conducted on a LightCycler^®^ 96 System (Roche, Basel, Switzerland), utilizing the *CmACT* gene (*MELO3C023264.1*) as the internal reference [19]. The qRT-PCR reaction mixture comprised 10 μL of Top Green qPCR SuperMix (TransGen Biotech), 0.5 μL each of forward and reverse primers (10 μmol·L^−1^, Table S1), 0.5 μL of cDNA template, and 8.5 μL of ddH_2_O, resulting in a final volume of 20 μL. The PCR program was configured as previously described [20] and each sample was run in triplicate. Relative gene expression levels were calculated as previously described [21].

## 3. Results

### 3.1. Overview of Metabolomic Data

**Figure 1:**
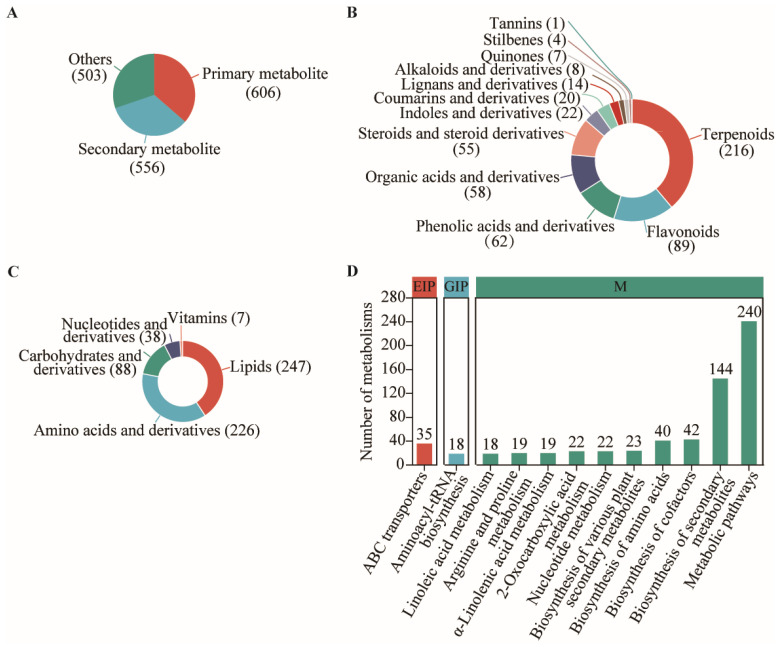
Phytochemical categorization of detected metabolites (**A**–**C**) and their corresponding KEGG functional pathways (**D**). EIP, environmental information processing; GIP, genetic information processing; M, metabolism.

To characterize adventitious root development in melon under waterlogging stress, hypocotyl tissues located beneath the water surface were subjected to TTC staining for morphological observation. The results showed that adventitious roots emerged at 72 h after waterlogging (Figure S1), followed by progressive elongation with prolonged stress exposure (Figure S2). For untargeted metabolomic quantification, hypocotyl samples were harvested at 0, 24, 48, and 72 h post-waterlogging stress. Principal component analysis (PCA) demonstrated clear discrimination among distinct sample groups (Figure S3), suggesting substantial divergence in their metabolic profiles. After data preprocessing and quality control, a total of 1430 metabolites were detected in positive ionization mode and 705 metabolites in negative ionization mode. Based on phytochemical classification, 606 metabolites were identified as primary metabolites, 556 as secondary metabolites, and 503 as other compounds (Figure 1A). Terpenoids constituted the dominant category of secondary metabolites (38.85%), followed by flavonoids (16.01%) and phenolic acids and derivatives (11.15%) (Figure 1B). Conversely, within the primary metabolite pool, lipids represented the most abundant class (40.76%), followed by amino acids and derivatives (37.29%), and carbohydrates and derivatives (14.52%) (Figure 1C).

The detected metabolites were functionally categorized according to the KEGG database. As illustrated in Figure 1D, these metabolites were annotated to three primary KEGG categories. Within the ‘environmental information processing’ category, the annotated pathway was ABC transporters. In the ‘genetic information processing’ category, the identified pathway was aminoacyl-tRNA biosynthesis. Furthermore, within the ‘metabolism’ category, the top priorities were metabolic pathways, followed by biosynthesis of secondary metabolites, biosynthesis of cofactors, biosynthesis of amino acids, and biosynthesis of various plant secondary metabolites.

### 3.2. Identification of Differentially Accumulated Metabolites (DAMs)

A total of 1337 DAMs were identified across all the pairwise comparisons (Table S2). Cluster analysis of these DAMs revealed five distinct change patterns (Figure S4). Specifically, there were 165, 427, 166, 330, and 249 metabolites in classes 1, 2, 3, 4, and 5, respectively. Metabolites in class 1 were significantly enriched in pathways related to arginine biosynthesis, alanine, aspartate and glutamate metabolism, ABC transporters, nicotinate and nicotinamide metabolism, nucleotide metabolism, cysteine and methionine metabolism, arginine and proline metabolism, D-amino acid metabolism, and aminoacyl-tRNA biosynthesis. Metabolites in class 2 were significantly enriched in nucleotide metabolism, α-linolenic acid metabolism, and purine metabolism. No pathways were significantly enriched for metabolites in class 3. Metabolites in class 4 were significantly enriched in nucleotide metabolism, while metabolites in class 5 were significantly enriched in α-linolenic acid metabolism (Table S3).

**Figure 2:**
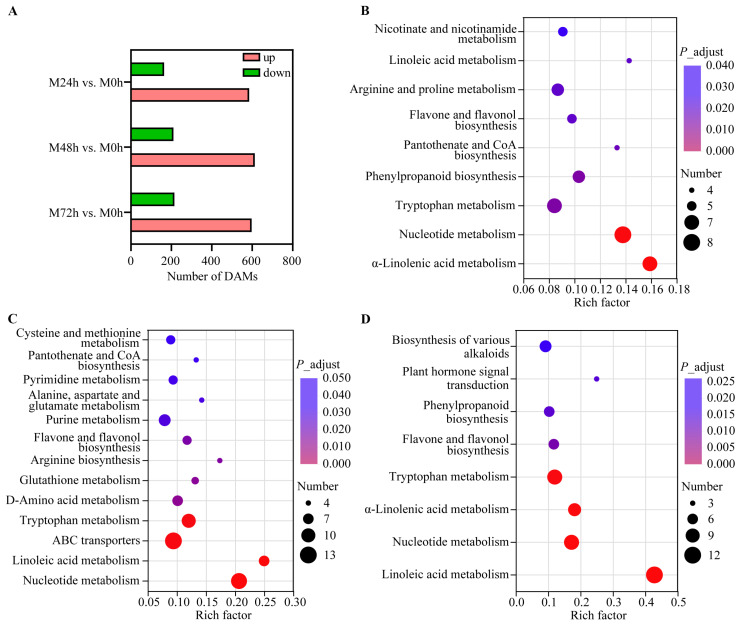
Characterization of differentially accumulated metabolites (DAMs). (**A**) Statistical histogram of DAMs. (**B**) KEGG enrichment analysis of the M24h vs. M0h comparison group. (**C**) KEGG enrichment analysis of the M48h vs. M0h comparison group. (**D**) KEGG enrichment analysis of the M72h vs. M0h comparison group.

In the M24h vs. M0h comparison group, a total of 749 DAMs were identified, comprising 585 up-regulated and 164 down-regulated metabolites (Figure 2A). These DAMs were significantly enriched in KEGG pathways of α-linolenic acid metabolism, nucleotide metabolism, tryptophan metabolism, phenylpropanoid biosynthesis, pantothenate and CoA biosynthesis, flavone and flavonol biosynthesis, arginine and proline metabolism, linoleic acid metabolism, and nicotinate and nicotinamide metabolism (Figure 2B). The M48h vs. M0h comparison group contained 823 DAMs, with 613 up-regulated and 210 down-regulated metabolites. These DAMs were notably enriched in KEGG pathways of nucleotide metabolism, linoleic acid metabolism, ABC transporters, tryptophan metabolism, D-amino acid metabolism, glutathione metabolism, arginine biosynthesis, flavone and flavonol biosynthesis, purine metabolism, alanine, aspartate and glutamate metabolism, pyrimidine metabolism, pantothenate and CoA biosynthesis, and cysteine and methionine metabolism (Figure 2C). In the M72h vs. M0h group, 597 metabolites were significantly up-regulated, while 215 were down-regulated. These DAMs were mainly enriched in linoleic acid metabolism, nucleotide metabolism, α-linolenic acid metabolism, tryptophan metabolism, flavone and flavonol biosynthesis, phenylpropanoid biosynthesis, plant hormone signal transduction, and biosynthesis of various alkaloids. Notably, pathways related to nucleotide metabolism, tryptophan metabolism, flavone and flavonol biosynthesis, and linoleic acid metabolism were enriched across all three comparison groups (Figure 2D).

**Figure 3:**
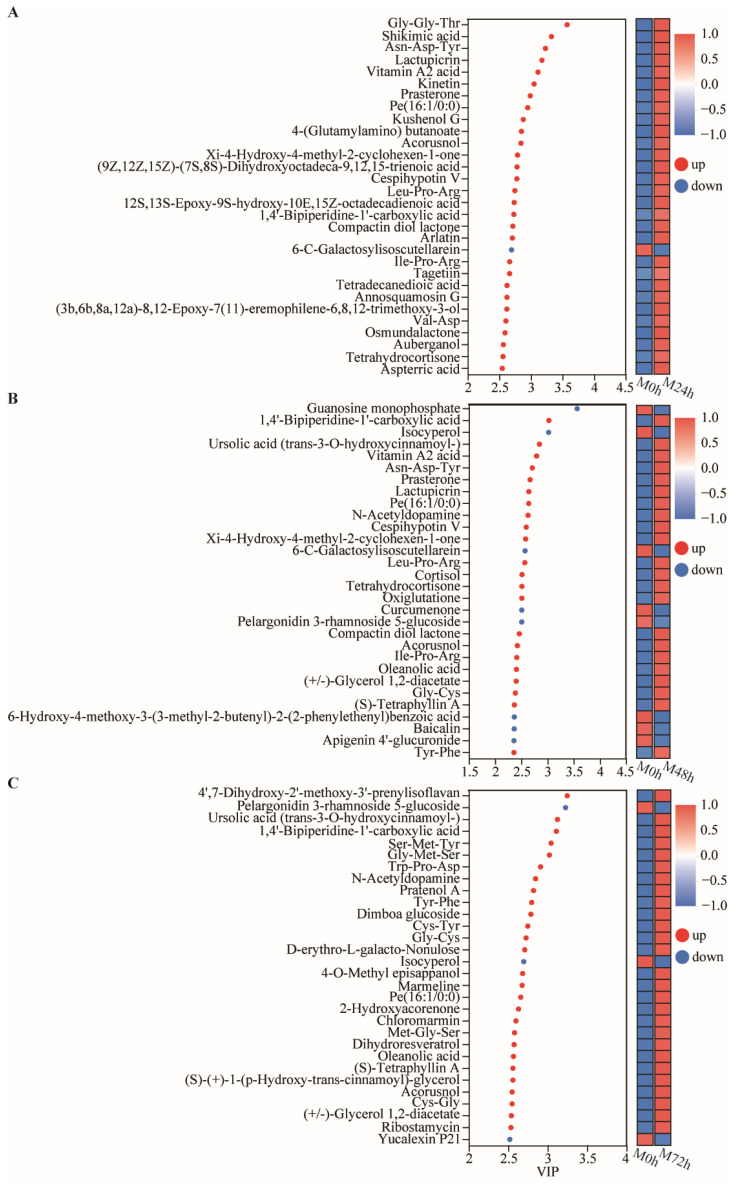
Metabolites with top variable importance in projection (VIP) scores in the M24h vs. M0h comparison group (**A**), M48h vs. M0h comparison group (**B**) and M72h vs. M0h comparison group (**C**).

In this study, the top 30 metabolites with the highest variable importance in projection (VIP) scores were detected across the comparison groups (Figure 3, Table S4). In the M24h vs. M0h comparison group, these metabolites were significantly enriched in three KEGG pathways, including phenylalanine, tyrosine and tryptophan biosynthesis, arginine and proline metabolism and biosynthesis of various alkaloids. In the M48h vs. M0h comparison group, the top 30 metabolites were significantly enriched in the KEGG pathway of glutathione metabolism. In the M72h vs. M0h comparison group, these metabolites were notably associated with two KEGG pathways, namely glutathione metabolism and neomycin, kanamycin and gentamicin biosynthesis. Among these metabolites, the accumulation of Pe(16:1/0:0), acorusnol, and 1,4′-bipiperidine-1′-carboxylic acid was significantly up-regulated at 24, 48, and 72 h after waterlogging stress compared with those at 0 h (Table S4). Acorusnol, categorized as a sesquiterpenoid, exhibited an increasing trend in accumulation during adventitious root formation induced by waterlogging stress, ranking second in up-regulation fold change in the M24h vs. M0h comparison group, third in the M48h vs. M0h group, and eleventh in the M72h vs. M0h group. 1,4′-bipiperidine-1′-carboxylic acid, a piperidine compound, ranked first in up-regulation fold change in both the M24h vs. M0h and M48h vs. M0h groups, and fifth in the M72h vs. M0h group; its VIP value ranked seventeenth in the M24h vs. M0h group, second in the M48h vs. M0h group, and fourth in the M72h vs. M0h group. 4′,7-dihydroxy-2′-methoxy-3′-prenylisoflavan, an isoflavone within the flavonoid class, was not identified as a differentially accumulated metabolite in the M24h vs. M0h and M48h vs. M0h comparison groups, but it ranked first in VIP value and second in up-regulation fold change in the M72h vs. M0h group (Tables S2 and S4).

### 3.3. Overview of Proteomic Data

**Figure 4:**
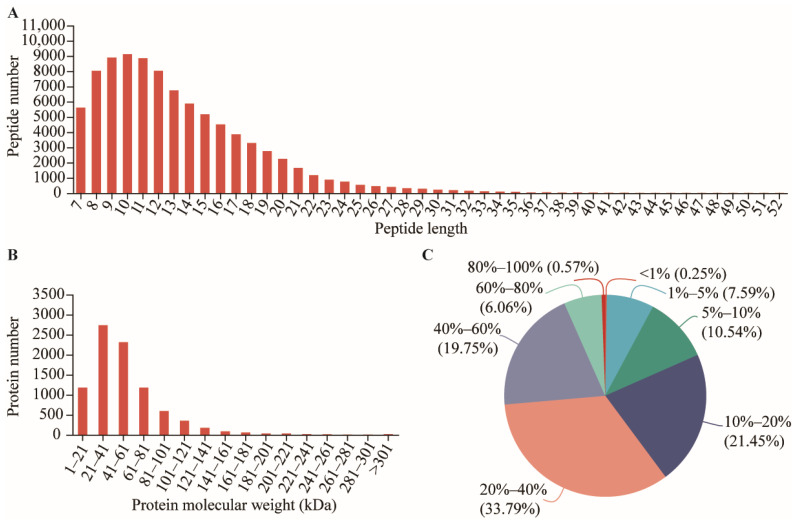
A survey of peptides and proteins. (**A**) Statistical histogram of peptides. (**B**) Statistical histogram of proteins. (**C**) Distribution of protein sequence coverage.

A total of 90,402 peptides and 8751 proteins were identified in this study. Statistical analysis of the identified peptide sequence lengths revealed that the majority of peptides ranged from 7 to 20 amino acids, accounting for 91.72% of all peptides (Figure 4A). The molecular weight of proteins was predominantly concentrated in the range of 21–61 kDa, with 5039 proteins representing 57.58% of the total. The numbers of proteins with molecular weights of 61–81 kDa, 1–21 kDa, and 81–101 kDa were 1175, 1174, and 590, respectively (Figure 4B). Proteins with peptide sequence coverage of 80–100%, 60–80%, 40–60%, 20–40%, 10–20%, and <10% accounted for 0.57%, 6.06%, 19.75%, 33.79%, 21.45%, and 18.38% of the total proteins, respectively (Figure 4C). These results indicate that most proteins exhibited favorable peptide sequence coverage, thereby supporting the accuracy and reliability of the proteomic data.

### 3.4. Identification of Differentially Expressed Proteins (DEPs)

**Figure 5:**
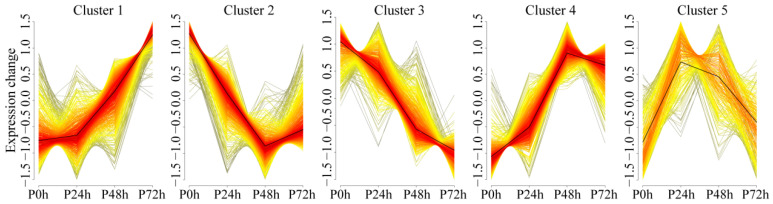
A cluster analysis of all differentially expressed proteins (DEPs).

Pairwise comparisons across different waterlogging treatment time points identified a total of 2898 DEPs (Table S5). Cluster analysis of all DEPs was conducted using the mFuzz package, which classified these proteins into five distinct expression clusters (Figure 5). Cluster 1 comprised 620 DEPs involved in phenylpropanoid biosynthesis, glutathione metabolism, α-linolenic acid metabolism, terpenoid backbone biosynthesis, pentose phosphate pathway, peroxidase activity, oxidoreductase activity, and reactive oxygen species metabolic process (Tables S6 and S7). Cluster 2 included 702 DEPs associated with photosynthesis, starch and sucrose metabolism, cyanoamino acid metabolism, glyoxylate and dicarboxylate metabolism, carbohydrate metabolic process, and brassinosteroid homeostasis. Cluster 3 contained 624 DEPs that participated in protein processing in endoplasmic reticulum, phagosome, ABC transporters, cutin, suberine and wax biosynthesis, protein complex oligomerization, and polysaccharide metabolic process. Cluster 4 included 700 DEPs involved in ribosome, transcription regulator activity, DNA-binding transcription factor activity, and multivesicular body. Finally, cluster 5 comprised 252 DEPs associated with rRNA processing, inorganic anion transmembrane transporter activity, cellular component biogenesis, secondary active transmembrane transporter activity, and ribonucleoprotein complex biogenesis (Tables S6 and S7).

**Figure 6:**
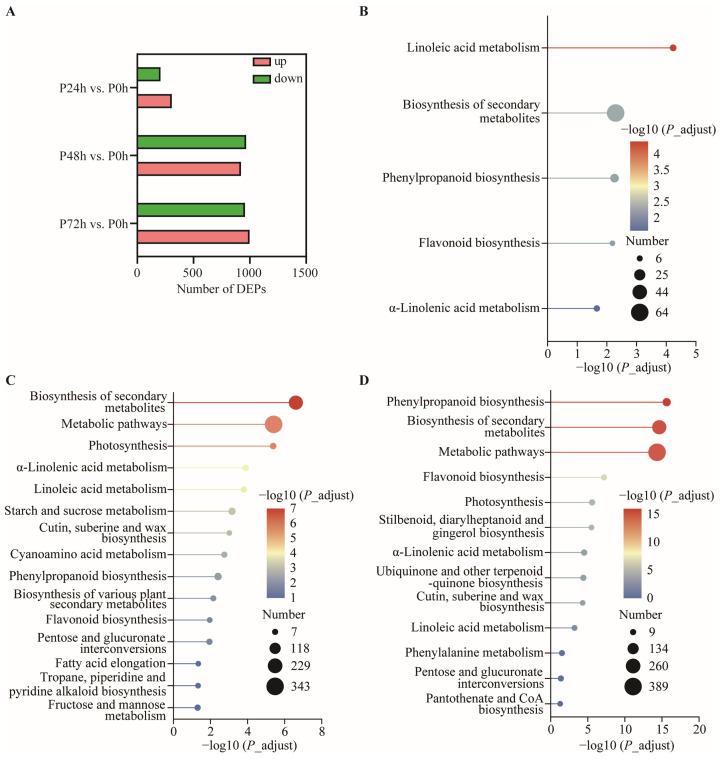
A survey of DEPs detected. (**A**) Statistical histogram of DEPs. (**B**) KEGG enrichment analysis of the P24h vs. P0h comparison group. (**C**) KEGG enrichment analysis of the P48h vs. P0h comparison group. (**D**) KEGG enrichment analysis of the P72h vs. P0h comparison group.

By normalizing the proteomic profiles of all samples to the P0h control group, dynamic variations in the number of DEPs were quantified. A distinct temporal response pattern was observed, with the number of DEPs exhibiting a gradual and continuous increase as the duration of waterlogging treatment was prolonged (Figure 6A). Compared to P0h, 307 DEPs were significantly up-regulated, while 207 were down-regulated at P24h. In the P48h vs. P0h comparison group, 922 DEPs were up-regulated and 967 were down-regulated. In the P72h vs. P0h comparison group, a total of 1955 DEPs were identified, comprising 998 up-regulated and 957 down-regulated proteins, which represented approximately 67.46% of the total DEPs (Figure 6A).

### 3.5. Enrichment Analysis of Identified DEPs

GO functional enrichment analysis of the DEPs identified 248, 195, and 211 significantly enriched GO terms in the P24h vs. P0h, P48h vs. P0h, and P72h vs. P0h comparison groups, respectively (Table S8). In the biological process category, the P24h vs. P0h comparison group was characterized by significant enrichment of terms such as monocarboxylic acid biosynthetic process, fatty acid biosynthetic process, oxylipin biosynthetic process, brassinosteroid homeostasis, phytosteroid metabolic process, phytosteroid biosynthetic process, and brassinosteroid biosynthetic process. The P48h vs. P0h comparison group highlighted significantly enriched biological processes, including response to stimulus, response to oxidative stress, carbohydrate metabolic process, protein complex oligomerization, oxylipin biosynthetic process, sterol metabolic process, steroid metabolic process, hydrogen peroxide catabolic process, and response to reactive oxygen species. For the P72h vs. P0h comparison group, the significantly enriched biological processes comprised secondary metabolic process, phenylpropanoid metabolic process, response to oxidative stress, secondary metabolite biosynthetic process, phenylpropanoid biosynthetic process, hydrogen peroxide catabolic process, lignin metabolic process, reactive oxygen species metabolic process, oxylipin biosynthetic process, and plant-type cell wall organization or biogenesis (Table S8).

KEGG pathway enrichment analysis of the DEPs revealed 5, 15, and 13 significantly enriched pathways in the comparison groups of P24h vs. P0h, P48h vs. P0h, and P72h vs. P0h, respectively (Figure 6B–D). Notably, linoleic acid metabolism, biosynthesis of secondary metabolites, phenylpropanoid biosynthesis, flavonoid biosynthesis, and α-linolenic acid metabolism were significantly enriched across all three pairwise comparisons. In addition, metabolic pathways, photosynthesis, cutin, suberine and wax biosynthesis, as well as pentose and glucuronate interconversions showed significant enrichment in both the P48h vs. P0h and P72h vs. P0h groups. In contrast, starch and sucrose metabolism, cyanoamino acid metabolism, biosynthesis of various plant secondary metabolites, fatty acid elongation, tropane, piperidine and pyridine alkaloid biosynthesis, as well as fructose and mannose metabolism were uniquely enriched in the P48h vs. P0h comparison group. Meanwhile, stilbenoid, diarylheptanoid and gingerol biosynthesis, ubiquinone and other terpenoid-quinone biosynthesis, phenylalanine metabolism, and pantothenate and CoA biosynthesis were specifically enriched only in the P72h vs. P0h comparison group.

### 3.6. Subcellular Localization of DEPs

**Figure 7:**
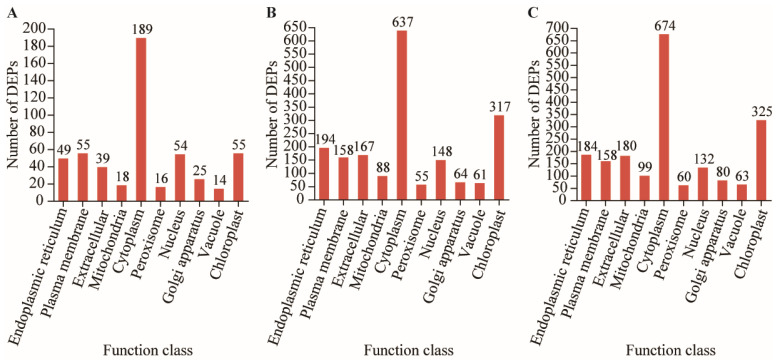
Subcellular localization analysis of the DEPs identified in the P24h vs. P0h comparison group (**A**), P48h vs. P0h comparison group (**B**) and P72h vs. P0h comparison group (**C**).

Subcellular localization prediction was performed for the DEPs identified in the comparison groups of P24h vs. P0h, P48h vs. P0h, and P72h vs. P0h. Across all three pairwise comparisons, DEPs were mapped to ten distinct subcellular compartments (Figure 7). In the P24h vs. P0h comparison group, 189 DEPs were localized to the cytoplasm, followed by 55 DEPs in the plasma membrane and 55 DEPs in the chloroplast. In the P48h vs. P0h comparison group, the majority of DEPs were localized to the cytoplasm (637 DEPs), with substantial proportions also detected in the chloroplast (317 DEPs) and the endoplasmic reticulum (194 DEPs). Similarly, in the P72h vs. P0h comparison group, the cytoplasm contained the largest number of DEPs (674 DEPs), with chloroplast (325 DEPs) and the endoplasmic reticulum (184 DEPs) representing other major localizations. Additionally, 54, 148, and 132 DEPs were localized to the nucleus in the P24h vs. P0h, P48h vs. P0h, and P72h vs. P0h comparison groups, respectively.

### 3.7. Analysis of DEPs Annotated as Transcription Factors

**Figure 8:**
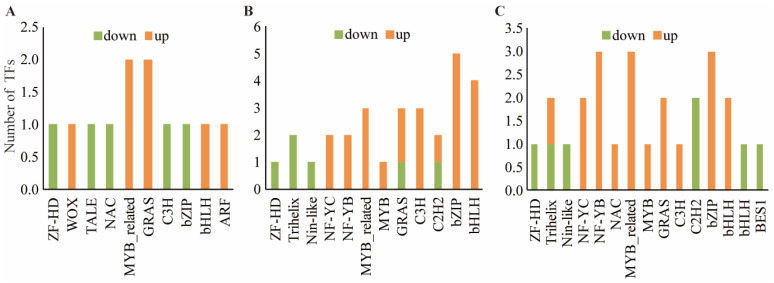
A survey of differentially expressed transcription factors detected in the P24h vs. P0h comparison group (**A**), P48h vs. P0h comparison group (**B**) and P72h vs. P0h comparison group (**C**).

To investigate the potential regulatory roles of transcription factors in waterlogging-induced adventitious root formation, DEPs were functionally categorized using the Plant Transcription Factor Database version 3.0 [22]. Across all pairwise comparisons, a total of 50 differentially expressed transcription factors were identified. Specifically, 12, 29, and 26 transcription factors were identified in the P24h vs. P0h, P48h vs. P0h, and P72h vs. P0h comparison groups, respectively (Figure 8). Among these transcription factors, MELO3C025904.1 (belonging to the GRAS family), MELO3C007640.1 (classified in the MYB-related family), and MELO3C022921.1 (assigned to the ZF-HD family) were concurrently detected in all three pairwise comparisons (Table S5). Notably, MELO3C007640.1 exhibited no expression at P0h, but its expression level was significantly up-regulated during adventitious root development induced by waterlogging stress. Furthermore, 6, 7, and 7 transcription factors were uniquely identified in the P24h vs. P0h, P48h vs. P0h, and P72h vs. P0h comparison groups, respectively. Specifically, the ARF family transcription factor MELO3C022932.1 was up-regulated by 1.62-fold in the P24h vs. P0h group, while the NF-YB family transcription factor MELO3C009309.1 exhibited a 4.80-fold up-regulation in the P72h vs. P0h group. Additionally, the bHLH family transcription factor MELO3C002197.1 was shared between the P48h vs. P0h and P72h vs. P0h comparison groups, with its expression level increasing by 219.33-fold and 381.29-fold in these two groups, respectively.

### 3.8. Identification of DEPs Participated in Adventitious Root Formation

Pyruvate decarboxylase (PDC) is a crucial enzyme involved in ethanol fermentation during anaerobic respiration in plants, catalyzing the conversion of pyruvate into acetaldehyde. The protein abundance of PDC, MELO3C009145.1 and MELO3C020531.1, was significantly up-regulated during adventitious rooting under waterlogging stress. Specifically, MELO3C009145.1 exhibited increases in protein abundance of 2.80-, 6.83-, and 5.49-fold, at 24, 48, and 72 h following waterlogging stress, respectively (Table S5). Similarly, alcohol dehydrogenase (ADH), another essential enzyme in ethanol fermentation, facilitates the conversion of acetaldehyde to ethanol. The expression of ADH, MELO3C023685.1 and MELO3C027151.1, was also markedly induced at 24, 48, and 72 h under waterlogging stress. The protein level of MELO3C023685.1 increased by 3.12-, 8.13-, and 7.01-fold, while MELO3C027151.1 demonstrated up-regulation of 2.61-, 8.04-, and 10.35-fold, respectively.

1-Aminocyclopropane-1-carboxylate oxidase (ACO) serves as the terminal key enzyme in the ethylene biosynthesis pathway in plants, catalyzing the oxidative decarboxylation of 1-aminocyclopropane-1-carboxylate, the ethylene precursor, to ultimately produce ethylene. We observed that the expression of ACO (MELO3C004619.1) was up-regulated by 9.61-, 109.67-, and 102.17-fold at 24, 48, and 72 h after waterlogging stress, respectively. Phenylalanine ammonia-lyase (PAL) functions as a crucial enzyme in salicylic acid biosynthesis, with MELO3C017810.1 being up-regulated by 1.54-, 3.05-, and 2.65-fold at 24, 48, and 72 h post waterlogging stress, respectively. Lipoxygenases encoded by MELO3C004247.1 and MELO3C004249.1, along with allene oxide synthases encoded by MELO3C010910.1 and MELO3C018413.1, which are involved in jasmonic acid biosynthesis, exhibited a down-regulated expression pattern during waterlogging stress-induced adventitious root formation (Table S5).

The results indicated that the protein levels of cell wall-related proteins, including extensin, pectinesterase, expansin, and polygalacturonase, underwent significant changes during adventitious root formation induced by waterlogging stress (Table S5). Notably, extensins MELO3C002087.1 and MELO3C008366.1, expansin MELO3C013295.1, and polygalacturonase MELO3C009970.1 were not expressed at 0 h of waterlogging stress but were significantly up-regulated at 24, 48, and 72 h following waterlogging treatment. The protein abundances of peroxidases (MELO3C014654.1, MELO3C018804.1, MELO3C025681.1 and MELO3C022435.1) were all significantly induced at 24, 48, and 72 h post-waterlogging stress. Among these peroxidases, MELO3C018804.1, MELO3C025681.1, and MELO3C022435.1 showed no expression at 0 h, whereas MELO3C014654.1 exhibited a relatively high basal expression level and was up-regulated by 2.19-, 3.66-, and 6.37-fold at 24, 48, and 72 h after waterlogging stress, respectively. Additionally, glutathione S-transferase encoded by MELO3C006220.1 was significantly up-regulated at 24, 48, and 72 h after waterlogging stress, while glutathione S-transferase MELO3C001978.1 and lactoylglutathione lyase MELO3C025360.1 were significantly up-regulated only at 48 and 72 h post-waterlogging treatment. Furthermore, cytochrome P450 proteins MELO3C023530.1 and MELO3C031255.1, as well as GDSL esterase/lipases MELO3C012919.1, MELO3C022690.1, MELO3C023647.1, and MELO3C012920.1, were all significantly up-regulated at 24, 48, and 72 h during adventitious rooting induced by waterlogging.

### 3.9. Integrative Analysis of Metabolome and Proteome

**Figure 9:**
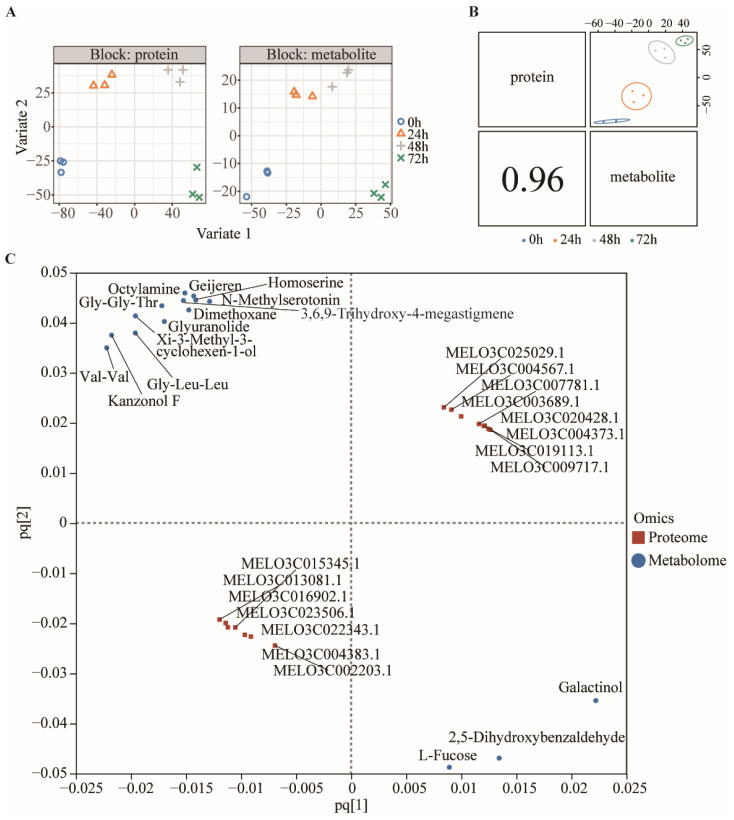
Integrative network analysis based on proteomic and metabolomic datasets. (**A**) Clear sample discrimination patterns exhibited by the proteomic (**left** panel) and metabolomic (**right** panel) profiles. (**B**) Pearson correlation analysis of the first principal components derived from proteomic and metabolomic data. (**C**) The top 15 influential proteins and metabolites based on O2PLS analysis.

To establish a comprehensive multiomic landscape of adventitious root formation under waterlogging stress, an integrated analysis of untargeted metabolomic and proteomic profiles was conducted using identical biological samples. As illustrated in Figure 9A, the scatter plots derived from the DIABLO model demonstrated distinct clustering of samples corresponding to different time points during waterlogging-induced adventitious rooting in both omic datasets. Additionally, strong correlations were observed between the latent components of the proteomic and metabolomic data, indicating a high degree of consistency and reliability of the constructed DIABLO model (Figure 9B). Furthermore, two-way orthogonal partial least squares (O2PLS) analysis was performed to investigate the inherent associations and cross-correlation between the proteome and metabolome profiles (Table S9). The top 15 proteins and metabolites exerting the greatest influence on the model based on O2PLS were presented in Figure 9C. The proteins with the most significant impact on the metabolome were associated with tryptophan metabolism, ascorbate and aldarate metabolism, biosynthesis of amino acids, carbon metabolism, biosynthesis of secondary metabolites, lipoic acid metabolism, and glutathione metabolism. The top 15 metabolites included three terpenoids, one indole and derivative, one flavonoid, two amino acids and derivatives, two oligopeptides, two carbohydrates and derivatives, one amine and derivative, one alcohol and derivative, one heterocyclic compound and one aldehyde and derivative. Among these metabolites, nine were differentially accumulated across the comparison groups, including Gly-Gly-Thr, 2,5-dihydroxybenzaldehyde, homoserine, L-fucose, galactinol, Gly-Leu-Leu, glyuranolide, Val-Val and N-methylserotonin.

Correlation analysis between the nine significant metabolites identified above and all DEPs revealed that 765 DEPs were significantly correlated with these metabolites (Table S10). These DEPs were associated with α-linolenic acid metabolism, flavonoid biosynthesis, fructose and mannose metabolism, starch and sucrose metabolism, and biosynthesis of secondary metabolites (Table S11). N-methylserotonin, classified as an indole and its derivative, exhibited significantly elevated accumulation in the M24h vs. M0h and M48h vs. M0h comparison groups (Table S2). A total of 105 DEPs were closely correlated with this metabolite, including one transcription factor from the ZF-HD family (MELO3C022921.1) and one from the GRAS family (MELO3C025904.1). While MELO3C022921.1 demonstrated a negative correlation with N-methylserotonin, MELO3C025904.1 exhibited a positive correlation. Additionally, MELO3C015961.1, which is involved in ethylene signal transduction, and MELO3C014795.1, implicated in glutathione metabolism, also showed positive correlations with N-methylserotonin. Val-Val, an amino acid and its derivative, displayed significantly increased accumulation across the M24h vs. M0h, M48h vs. M0h, and M72h vs. M0h comparison groups. In total, 574 DEPs were closely associated with Val-Val, encompassing transcription factors from the bZIP (MELO3C013408.1), C2H2 (MELO3C009430.1), GRAS (MELO3C025904.1), and bHLH (MELO3C017669.1) families. Among these, MELO3C013408.1, MELO3C025904.1, and MELO3C017669.1 were positively correlated with Val-Val, while MELO3C009430.1 displayed a negative correlation. Glyuranolide, a terpenoid, showed significantly enhanced accumulation in the M24h vs. M0h and M48h vs. M0h comparison groups. A total of 156 DEPs were tightly correlated with glyuranolide, including transcription factors from the ZF-HD (MELO3C022921.1) and GRAS (MELO3C025904.1) families, as well as LRR receptor-like serine/threonine-protein kinase (MELO3C002389.1) and polygalacturonase (MELO3C009970.1). MELO3C002389.1, MELO3C025904.1, and MELO3C009970.1 exhibited positive correlations with glyuranolide, whereas MELO3C022921.1 showed a negative correlation. Collectively, these identified metabolites and proteins are presumed to modulate adventitious root formation in melon under waterlogging stress, yet detailed functional mechanisms remain to be experimentally validated.

### 3.10. Quantitative Reverse Transcription-PCR (qRT-PCR) Validation

To further verify the reliability of the proteomic data, nine representative DEPs were selected to assess their relative transcript levels by qRT-PCR. The functional proteins encoded by these candidate genes included alcohol dehydrogenase, peroxidase, extensin, 1-aminocyclopropane-1-carboxylate oxidase, and various transcription factors. The qRT-PCR results revealed that these genes exhibited obvious expression differences in the process of waterlogging-triggered adventitious rooting (Figure S5). Importantly, the transcript expression trends closely aligned with the accumulation patterns of their corresponding proteins identified through proteomic analysis.

## 4. Discussion

Waterlogging represents one of the most prevalent and destructive abiotic stresses in global agricultural systems. It markedly disrupts plant growth and development, and poses a major constraint to crop yield and quality. To mitigate the detrimental impacts of waterlogging, higher plants have evolved distinct adaptive mechanisms during long-term natural evolution. Accumulating evidence has demonstrated that adventitious root formation is an innate evolutionary strategy widespread in wild plant species. The cultivated agricultural crops inherit this waterlogging-adaptive trait, which is further strengthened by artificial domestication, to withstand waterlogging stress [8,12]. The formation of adventitious roots is a complex developmental process, which is precisely regulated by both internal and external factors [23]. It is generally believed that the development of adventitious roots undergoes an induction stage, initiation stage and expression stage [24]. Adventitious roots contain advanced aerenchyma and can obtain oxygen from the air directly, which is beneficial for coping with hypoxic environments caused by waterlogging [25]. As a species characterized by an inherently taproot-dominant root architecture, melon seedlings scarcely generate spontaneous adventitious roots under well-drained normal growth conditions. Upon exposure to waterlogging stress, seedlings undergo extensive morphological and physiological remodeling, accompanied by prominent adventitious root formation on the hypocotyls [7]. In the present study, we aimed to investigate the response mechanism of adventitious rooting in melon under waterlogging stress at the protein and metabolic levels.

Adventitious roots were initiated on the hypocotyls of melon seedlings upon waterlogging treatment, with visible root protrusion observed at 72 h post-waterlogging (Figure S1). In our previous report, the developmental process of adventitious roots in melon was divided into three stages, including the induction stage (0–24 h after waterlogging), the initiation stage (24–48 h after waterlogging), and the expression stage (48–72 h after waterlogging) [7]. In the present study, to explore the molecular mechanism of adventitious rooting, we conducted proteomic and metabolomic analyses on melon hypocotyl samples collected at 0, 24, 48, and 72 h post-waterlogging. The results revealed that compared with 0 h, a total of 514, 1889 and 1955 differentially expressed proteins (DEPs) were identified at 24, 48 and 72 h after waterlogging, respectively (Figure 6A), while 749, 823 and 812 differentially accumulated metabolites (DAMs) were detected at the corresponding time points (Figure 2A). These findings indicated that the numbers of DEPs and DAMs were higher at 48 and 72 h post-waterlogging compared to 24 h. This observation aligns with the previously reported transcriptomic results of adventitious rooting in melon [7], further demonstrating that significant changes at the protein and metabolic levels occur during the initiation and expression stages of adventitious root formation. In this study, as the duration of waterlogging stress extended, continuous elongation of adventitious roots was clearly detected at 7 days after waterlogging (Figure S2). Previous research has demonstrated that the removal of adventitious roots triggers severe abnormal phenotypes including leaf chlorosis and wilting under waterlogging conditions [14], highlighting the irreplaceable function of these waterlogging-induced adventitious roots. Nevertheless, even with abundant adventitious roots formed upon prolonged waterlogging exposure, melon seedling growth was significantly suppressed, alongside a pronounced reduction in leaf chlorophyll content [14]. Collectively, these findings suggest that adventitious roots cannot fully offset waterlogging damage, and the formation of adventitious roots is a survival-oriented emergency adaptation under waterlogging stress.

Under soil waterlogging, rapid water saturation of soil pores drastically reduces oxygen availability around plant roots due to the extremely low oxygen diffusion rate in water, leading to root hypoxia [26]. Previous studies have proven that plants have evolved sophisticated adaptive mechanisms against hypoxia. A core adaptive strategy is to reprogram respiratory metabolism, whereby efficient aerobic respiration is shifted to anaerobic respiration. This metabolic transition partially compensates for energy deficiency under oxygen limitation and sustains fundamental vital activities [27,28,29]. Pyruvate decarboxylase (PDC) and alcohol dehydrogenase (ADH) are two key enzymes involved in the ethanolic fermentation pathway of plant anaerobic respiration. PDC catalyzes the decarboxylation of pyruvate to acetaldehyde, while ADH further reduces acetaldehyde to ethanol using NADH as an electron donor, accompanied by the regeneration of NAD^+^. This process not only prevents cytotoxicity caused by excessive acetaldehyde accumulation, but also maintains the continuous operation of glycolysis, thereby supplying basal energy for plants under hypoxic stress [30,31]. In the present study, proteomic analysis revealed that the protein abundances of PDC (MELO3C009145.1 and MELO3C020531.1) and ADH (MELO3C023685.1 and MELO3C027151.1) were significantly up-regulated at 24, 48 and 72 h post waterlogging (Table S5). These protein expression patterns are highly consistent with our previous transcriptomic findings, in which the genes encoding PDC and ADH were transcriptionally induced during adventitious root formation in waterlogged melon seedlings [7]. Similarly, a proteomic study on adventitious root development in waterlogged cucumber seedlings also reported pronounced increases in the abundance of PDC and ADH [32]. Collectively, these results suggest that hypoxia induced by waterlogging activates the ethanolic fermentation pathway of anaerobic respiration in melon seedlings, which provides basic energy for adventitious root development. In turn, newly formed adventitious roots effectively alleviate hypoxic stress in root systems. This regulatory mechanism plays an indispensable role in the survival and stress tolerance of melon seedlings exposed to waterlogging.

Ethylene serves as an essential endogenous gaseous hormone that orchestrates plant growth, developmental progression, and stress adaptation. This phytohormone participates extensively in multiple fundamental physiological processes, ranging from seed germination and seedling morphogenesis to floral sex differentiation and fruit ripening. Additionally, ethylene is indispensable for plant acclimation to diverse abiotic stressors, including salinity, drought, waterlogging and extreme temperature conditions [33,34,35]. Accumulating evidence has demonstrated a conserved regulatory role of ethylene in modulating adventitious root development across plant species. Its positive regulatory capacity for adventitious root formation has been experimentally validated in a variety of plant species, such as mung bean [36], marigold [37], cucumber [38], tomato [39], petunia [40], and rice [41]. Upon waterlogging exposure, hypoxia rapidly induces ethylene biosynthesis and accumulation in submerged plant tissues, a critical signaling event that mediates adventitious root formation [42,43,44,45]. As a key rate-limiting enzyme governing the ethylene biosynthesis cascade, 1-aminocyclopropane-1-carboxylate oxidase (ACO) directly determines ethylene production efficiency under hypoxic conditions via its expression level [46]. A proteomic study of cucumber revealed that during waterlogging-induced adventitious root formation, ACO protein abundance was significantly up-regulated, accompanied by a concomitant increase in ethylene content in the hypocotyl, which directly validated the role of ACO-mediated ethylene biosynthesis in adventitious root formation [32]. In the present study, the protein product of the melon ACO-encoding gene MELO3C004619.1 was found to be continuously and substantially up-regulated at 24, 48 and 72 h after waterlogging stress (Table S5). This result is highly consistent with findings in cucumber, further revealing the functional conservation of ACO in waterlogging responses among Cucurbitaceae crops. In addition to ethylene biosynthesis, ethylene signal perception also participates in regulating adventitious root development under waterlogging stress. MELO3C015961.1, annotated as a putative ethylene response sensor 1 (ERS1), exhibited markedly higher protein abundance at 24 h and 48 h after waterlogging compared with the 0 h control in our proteomic data (Table S5). As a typical membrane-localized ethylene receptor, ERS1 perceives accumulated ethylene signals on the plasma membrane and initiates downstream ethylene signal transduction cascades. Combined with the elevated ACO protein level (MELO3C004619.1) that accelerates ethylene synthesis, the up-regulation of ERS1 protein further suggests that both ethylene production and ethylene sensing are synchronously activated in the process of waterlogging-triggered adventitious rooting. Given the positive correlation between adventitious root formation and plant waterlogging tolerance [12,15,47], we hypothesize that the ACO encoded by *MELO3C004619.1* and ERS1 encoded by *MELO3C015961.1* may jointly trigger downstream transcriptional events responsible for adventitious rooting under hypoxic conditions. Further functional validation of these two genes using genetic approaches will be conducted in subsequent studies, aiming to provide a novel target for elucidating the molecular mechanism of waterlogging adaptation in melon.

Transcription factors serve as master regulators that activate or repress gene expression, playing critical roles in plant adaptation to abiotic stresses [48,49]. Previous studies have documented that transcription factors including MYB, WRKY, bHLH, GRAS, ZF-HD, ARF, LBD, C2H2, ERF and NAC families participate in plant responses to waterlogging stress [50,51]. In the present study, a total of 50 differentially expressed transcription factors were identified to be involved in waterlogging–induced adventitious root formation (Table S5). Notably, three key transcription factors, namely the GRAS family protein MELO3C025904.1, MYB-related protein MELO3C007640.1, and ZF-HD family protein MELO3C022921.1, were consistently differentially expressed in the P24h vs. P0h, P48h vs. P0h, and P72h vs. P0h comparison groups, suggesting their essential and persistent regulatory roles throughout the entire process of waterlogging-triggered adventitious root development in melon. GRAS and ZF-HD transcription factors are well-established regulators of plant root architecture remodeling, cell proliferation, and abiotic stress signal transduction in horticultural crops [52,53,54,55]. Their continuous differential expression in this study reinforces their conserved functions in linking hypoxic stress signaling and adventitious root developmental adaptation in melon. Importantly, MELO3C007640.1 (MYB-related family) was completely undetectable under normal conditions but was significantly up-regulated upon waterlogging treatment, exhibiting a strictly stress-induced expression pattern. This finding strongly suggests that MELO3C007640.1 acts as a pivotal positive regulator specifically activated by waterlogging hypoxia signals, and is tightly associated with the initiation and sustained outgrowth of adventitious roots in melon seedlings. At the late-stress stage, the significant induction of the NF-YB family protein MELO3C009309.1 indicates that NF-YB members participate in maintaining sustained root growth and enhancing long-term waterlogging tolerance under prolonged hypoxic conditions. Moreover, the bHLH family protein MELO3C002197.1, shared by the P48h vs. P0h and P72h vs. P0h groups, displayed a striking and continuously elevated expression level (Table S5). As a large and functionally diverse transcription factor family in plants, bHLH proteins are central integrators of hypoxic signal transduction and plant adaptive growth [56,57]. The progressively amplified up-regulation of MELO3C002197.1 with prolonged stress duration indicates that this bHLH member serves as a highly sensitive positive regulator predominantly functioning in the middle-to-late stage of adventitious root development, potentially facilitating hypoxia adaptation by robustly activating downstream stress-defensive and root-developmental gene expression.

Global metabolic reprogramming is another core adaptive feature in response to waterlogging [58]. In this study, we identified a total of 2135 metabolites, which were classified into primary metabolites, secondary metabolites and other compounds (Figure 1A). Lipids were the most abundant primary metabolites (Figure 1C), and pathways related to linoleic acid metabolism and α-linolenic acid metabolism were persistently enriched during adventitious rooting (Figure 2B–D), which was in accord with proteomic analysis (Figure 6B–D). It has been reported that linoleic acid and α-linolenic acid are essential unsaturated fatty acids in plants, which are not only important components of cell membranes but also serve as precursors for the synthesis of oxylipins [59]. Oxylipin-mediated signaling pathways are widely involved in hypoxia response, cell wall remodeling and root morphogenesis [60,61]. Proteomic profiling revealed significant enrichment of the oxylipin biosynthetic process in P24h vs. P0h, P48h vs. P0h and P72h vs. P0h comparison groups (Table S8), indicating that oxylipin-mediated signaling pathways were involved in the process of adventitious rooting induced by waterlogging. Terpenoids, a group of plant compounds with antioxidant and stress-protective functions, yielded two key metabolites linked to flooding stress response in watermelon [58]. In this study, we found a sesquiterpenoid, acorusnol, displayed a sustained up-regulation trend during waterlogging treatment, ranking among the top metabolites with high VIP values and fold changes across all comparison groups (Table S4), implying that terpenoid metabolism was persistently activated to eliminate excess ROS generated by anaerobic respiration and oxidative stress, so as to guarantee the division and differentiation of hypocotyl cells for adventitious root formation. Piperidine alkaloids are closely associated with plant stress resistance [62,63,64]. We found 1,4′-bipiperidine-1′-carboxylic acid, a piperidine alkaloid, exhibited extremely high fold changes in accumulation at 24 h and 48 h, and maintained a high expression level at 72 h (Table S4), which demonstrated that alkaloid metabolism is a unique adaptive pathway activated during the initiation and elongation of adventitious roots, and piperidine compounds may function as key signal molecules or protective metabolites linking hypoxic stress response and root morphogenesis.

Multi-omics integrated analysis is an effective strategy to unravel the complex molecular regulatory networks of plant stress responses and developmental processes [65]. In this study, DIABLO analysis confirmed a strong correlation between the proteomic and metabolomic datasets (Figure 9), proving that the changes in protein abundance and metabolite accumulation were highly coordinated during adventitious root formation under waterlogging in melon. Correlation analysis further screened nine core differential metabolites closely linked to hundreds of DEPs, covering amino acid derivatives, terpenoids, indole derivatives, carbohydrates and oligopeptides (Table S10). Glutathione metabolism, a classic antioxidant pathway, was jointly enriched in both omics datasets. Multiple glutathione S-transferases and lactoylglutathione lyase were significantly up-regulated at the protein level, while metabolites related to glutathione metabolism also accumulated dynamically (Figure 2, Table S5). The significant correlation between glutathione-related DEPs and core metabolites such as N-methylserotonin indicated that the glutathione antioxidant system forms a joint regulatory network with small-molecule metabolites to jointly scavenge ROS and relieve oxidative damage under long-term hypoxia. As a key indole derivative, N-methylserotonin was significantly accumulated at the early and middle stages of waterlogging, and it had close correlations with two pivotal transcription factors (GRAS family MELO3C025904.1 and ZF-HD family MELO3C022921.1) that were continuously differentially expressed in P24h vs. P0h, P48h vs. P0h and P72h vs. P0h comparison groups (Tables S2 and S10). GRAS and ZF-HD transcription factors are central regulators of plant root development and stress signal transduction [52,53,54,55]. The positive correlation between GRAS protein and N-methylserotonin, as well as the negative correlation between ZF-HD protein and this metabolite (Table S10), suggested that N-methylserotonin may act as an intermediate signal molecule to regulate the transcriptional activity of transcription factors, thereby linking hypoxic stress signals to the downstream developmental programs of adventitious roots. Val-Val, a dipeptide derived from amino acid metabolism, was continuously up-regulated during adventitious rooting and correlated with the largest number of DEPs, including multiple bZIP, C2H2, GRAS and bHLH transcription factors (Tables S2 and S10). Amino acid-derived oligopeptides can not only serve as nitrogen nutrition reserves for cell growth, but also function as signal molecules to participate in plant stress response and morphogenesis [66,67]. The extensive correlation between Val-Val and various transcription factors and functional proteins implied that amino acid metabolism and its derivative peptides occupy a core position in the regulatory network of adventitious root formation under waterlogging in melon. In addition, the terpenoid metabolite glyuranolide was significantly correlated with cell wall metabolism-related proteins such as polygalacturonase and receptor-like kinases (Table S10). Polygalacturonase is a key enzyme involved in cell wall degradation and remodeling, which is a prerequisite for the division and elongation of root primordia [68,69,70,71]. This correlation further established the connection between terpenoid metabolism and cell wall modification, explaining how secondary metabolites participate in regulating the morphological development of adventitious roots.

## 5. Limitations of the Study

This work dissected metabolomic and proteomic regulatory cascades governing adventitious root formation, which acts as a primary adaptive strategy for *Cucumis melo* against waterlogging stress. Nevertheless, waterlogging tolerance in plants is a complex and multidimensional trait that cannot be completely explained merely by the development of adventitious roots. Synergistic rhizosphere microbial crosstalk and systemic physiological reprogramming are also indispensable regulatory modules that facilitate plant acclimation to waterlogging. Waterlogging drastically remodels the rhizosphere microenvironment by triggering severe soil hypoxia, which can reshape the community composition, relative abundance, and metabolic functionality of root-associated microbiota. The waterlogging tolerance of melon might be a comprehensive result of morphological adaptation (adventitious root formation), rhizosphere microbial interaction, and systemic physiological regulation. Future research will integrate microbiome sequencing approaches to explore the multilayered microbe–physiology–morphology regulatory network associated with melon waterlogging tolerance, thereby comprehensively unraveling the synergistic adaptive mechanisms under waterlogging conditions.

## 6. Conclusions

In summary, the present study systematically dissects the molecular regulatory network underlying waterlogging acclimation via adventitious root development in melon, through integrated proteomic and metabolomic analyses. The identified core DAMs, DEPs, and vital metabolic pathways constitute a valuable resource of candidate molecules to facilitate the genetic improvement of waterlogging-resistant melon germplasm. However, the detailed molecular crosstalk between key metabolites and regulatory proteins, as well as the intrinsic physiological roles of master transcription factors and metabolic enzymes, require in-depth functional characterization through genetic manipulation and exogenous metabolite supplementation in future investigations.

## Data Availability

All datasets obtained for this study are included in the manuscript/Supplementary Data.

## Supplementary Materials

The following supporting information can be downloaded at: https://www.mdpi.com/article/10.3390/biology15141185/s1, Figure S1: Morphological observation of adventitious root development in melon under waterlogging stress. (A) Photograph of hypocotyl at 0 h after waterlogging (HAW); (B) 24 HAW; (C) 48 HAW; (D) 72 HAW; (E) 96 HAW. Bars, 5 mm. Figure S2: Morphological phenotypes of melon seedlings photographed 7 days after waterlogging stress. Bars, 1 cm. Figure S3: Principal component analysis (PCA) plots of untargeted metabolomic data. Figure S4: A cluster analysis of all differentially accumulated metabolites (DAMs). Figure S5: Quantitative reverse transcription-PCR (qRT-PCR) verification of some key genes in the proteome. The histograms denote qRT-PCR results and line graphs represent proteomic data. Table S1: The primer sequences for quantitative reverse transcription-PCR (qRT-PCR) verification. Table S2: The detailed information of differentially accumulated metabolites (DAMs). Table S3: KEGG enrichment analysis of five clusters in DAMs. Table S4: Detailed information of the top 30 metabolites with variable importance in projection (VIP) scores across the comparison groups. Table S5: The detailed information of differentially expressed proteins (DEPs). Table S6: KEGG enrichment analysis of five clusters in DEPs. Table S7: GO enrichment analysis of five clusters in DEPs. Table S8: GO enrichment analysis of DEPs in three groups compared to the control. Table S9: The O2PLS analysis between proteome and metabolome. Table S10: Correlation network analysis between nine significant metabolites and DEPs. Table S11: KEGG enrichment analysis of 765 DEPs.

## References

[1] Fukao T., Barrera-Figueroa B.E., Juntawong P., Peña-Castro J.M. (2019). Submergence and waterlogging stress in plants: A review highlighting research opportunities and understudied aspects. Front. Plant Sci..

[2] Chen K., Hu Q., Ma X., Zhang X., Qian R., Zheng J. (2024). The effect of exogenous melatonin on waterlogging stress in *Clematis*. Front. Plant Sci..

[3] Zhou W., Chen F., Meng Y., Chandrasekaran U., Luo X., Yang W., Shu K. (2020). Plant waterlogging/flooding stress responses: From seed germination to maturation. Plant Physiol. Biochem..

[4] Ahsan N., Lee D.G., Lee S.H., Lee K.W., Bahk J.D., Lee B.H. (2007). A proteomic screen and identification of waterlogging-regulated proteins in tomato roots. Plant Soil.

[5] Manghwar H., Hussain A., Alam I., Khoso M.A., Ali Q., Liu F. (2024). Waterlogging stress in plants: Unraveling the mechanisms and impacts on growth, development, and productivity. Environ. Exp. Bot..

[6] Zhong Y.H., Guo Z.J., Wei M.Y., Wang J.C., Song S.W., Chi B.J., Zhang Y.C., Liu J.W., Li J., Zhu X.Y. (2023). Hydrogen sulfide upregulates the alternative respiratory pathway in mangrove plant *Avicennia marina* to attenuate waterlogging-induced oxidative stress and mitochondrial damage in a calcium-dependent manner. Plant Cell Environ..

[7] Zhang H.X., Li G.Q., Yan C.P., Cao N., Yang H.D., Le M.W., Zhu F.H. (2021). Depicting the molecular responses of adventitious rooting to waterlogging in melon hypocotyls by transcriptome profiling. 3 Biotech.

[8] Koramutla M.K., Tuan P.A., Ayele B.T. (2022). Salicylic acid enhances adventitious root and aerenchyma formation in wheat under waterlogged conditions. Int. J. Mol. Sci..

[9] Liang K., Tang K., Fang T., Qiu F. (2020). Waterlogging tolerance in maize: Genetic and molecular basis. Mol. Breed..

[10] Wu J., Cheng J., Xu C., Qi S., Sun W., Wu S. (2020). AUREA maintains the balance between chlorophyll synthesis and adventitious root formation in tomato. Hortic. Res..

[11] Peng Y., Zhu J., Li W., Gao W., Shen R., Meng L. (2020). Effects of grafting on root growth, anaerobic respiration enzyme activity and aerenchyma of bitter melon under waterlogging stress. Sci. Hortic..

[12] Pan J., Song J., Sohail H., Sharif R., Yan W., Hu Q., Qi X., Yang X., Xu X., Chen X. (2024). RNA-seq-based comparative transcriptome analysis reveals the role of *CsPrx73* in waterlogging-triggered adventitious root formation in cucumber. Hortic. Res..

[13] Shimamura S., Yoshida S., Mochizuki T. (2007). Cortical aerenchyma formation in hypocotyl and adventitious roots of *Luffa cylindrica* subjected to soil flooding. Ann. Bot..

[14] Zhang H., Yan C., Chen Q., Li G. (2025). Depicting the physiological, biochemical and metabolic responses to the removal of adventitious roots and their functions in *Cucumis melo* under waterlogging stress. Agronomy.

[15] Steffens B., Rasmussen A. (2016). The physiology of adventitious roots. Plant Physiol..

[16] Mui N.T., Zhou M., Parsons D., Smith R.W. (2021). Aerenchyma formation in adventitious roots of tall fescue and cocksfoot under waterlogged conditions. Agronomy.

[17] Wang X., Li X., Deng X., Han H., Shi W., Li Y. (2007). A protein extraction method compatible with proteomic analysis for the euhalophyte *Salicornia europaea*. Electrophoresis.

[18] Chen Q., Liang X., Wu T., Jiang J., Jiang Y., Zhang S., Ruan Y., Zhang H., Zhang C., Chen P. (2022). Integrative analysis of metabolomics and proteomics reveals amino acid metabolism disorder in sepsis. J. Transl. Med..

[19] Kong Q., Yuan J., Niu P., Xie J., Jiang W., Huang Y., Bie Z. (2014). Screening suitable reference genes for normalization in reverse transcription quantitative real-time PCR analysis in melon. PLoS ONE.

[20] Zhang H., Li G., Cao N., Yang H., Zhu F. (2021). Genome-wide identification and expression analysis of NRAMP transporter genes in *Cucumis sativus* and *Citrullus lanatus*. Can. J. Plant Sci..

[21] Livak K.J., Schmittgen T.D. (2001). Analysis of relative gene expression data using real-time quantitative PCR and the 2^−ΔΔCT^ method. Methods.

[22] Jin J., Zhang H., Kong L., Gao G., Luo J. (2014). PlantTFDB 3.0: A portal for the functional and evolutionary study of plant transcription factors. Nucleic Acids Res..

[23] da Costa C.T., de Almeida M.R., Ruedell C.M., Schwambach J., Maraschin F.S., Fett-Neto A.G. (2013). When stress and development go hand in hand: Main hormonal controls of adventitious rooting in cuttings. Front. Plant Sci..

[24] Li S.W., Xue L.G., Xu S.J., Feng H.Y., An L.Z. (2009). Mediators, genes and signaling in adventitious rooting. Bot. Rev..

[25] Xu X., Ji J., Xu Q., Qi X., Chen X. (2017). Inheritance and quantitative trail loci mapping of adventitious root numbers in cucumber seedlings under waterlogging conditions. Mol. Genet. Genom..

[26] Jackson M.B. (1985). Ethylene and responses of plants to soil waterlogging and submergence. Annu. Rev. Plant Physiol..

[27] Zhang Y., Liang T., Dong H. (2024). Melatonin enhances waterlogging tolerance of field-grown cotton through quiescence adaptation and compensatory growth strategies. Field Crop. Res..

[28] Ma S., Gai P., Geng B., Wang Y., Ullah N., Zhang W., Zhang H., Fan Y., Huang Z. (2022). Exogenous melatonin improves waterlogging tolerance in wheat through promoting antioxidant enzymatic activity and carbon assimilation. Agronomy.

[29] Gu X., Xue L., Lu L., Xiao J., Song G., Xie M., Zhang H. (2021). Melatonin enhances the waterlogging tolerance of *Prunus persica* by modulating antioxidant metabolism and anaerobic respiration. J. Plant Growth Regul..

[30] Ahmad S., Wang G.Y., Muhammad I., Zeeshan M., Zhou X.B. (2022). Melatonin and KNO_3_ application improves growth, physiological and biochemical characteristics of maize seedlings under waterlogging stress conditions. Biology.

[31] Li C., Bai T., Ma F., Han M. (2010). Hypoxia tolerance and adaptation of anaerobic respiration to hypoxia stress in two *Malus* species. Sci. Hortic..

[32] Xu X., Ji J., Ma X., Xu Q., Qi X., Chen X. (2016). Comparative proteomic analysis provides insight into the key proteins involved in cucumber (*Cucumis sativus* L.) adventitious root emergence under waterlogging stress. Front. Plant Sci..

[33] Khan N.A., Khan M.I.R., Ferrante A., Poor P. (2017). Ethylene: A key regulatory molecule in plants. Front. Plant Sci..

[34] Alves L.R., Monteiro C.C., Carvalho R.F., Ribeiro P.C., Tezotto T., Azevedo R.A., Gratão P.L. (2017). Cadmium stress related to root-to-shoot communication depends on ethylene and auxin in tomato plants. Environ. Exp. Bot..

[35] Ribeiro R.P., Costa L.C., Medina E.F., Araújo W.L., Zsögön A., Ribeiro D.M. (2018). Ethylene coordinates seed germination behavior in response to low soil pH in *Stylosanthes humilis*. Plant Soil.

[36] Pan R., Wang J., Tian X. (2002). Influence of ethylene on adventitious root formation in mung bean hypocotyl cuttings. Plant Growth Regul..

[37] Jin X., Liao W., Yu J., Ren P., Dawuda M.M., Wang M., Niu L., Li X., Xu X. (2017). Nitric oxide is involved in ethylene-induced adventitious rooting in marigold (*Tagetes erecta* L.). Can. J. Plant Sci..

[38] Huang D., Bian B., Zhang M., Wang C., Li C., Liao W. (2020). The role and proteomic analysis of ethylene in hydrogen gas-induced adventitious rooting development in cucumber (*Cucumis sativus* L.) explants. PeerJ.

[39] Negi S., Sukumar P., Liu X., Cohen J.D., Muday G.K. (2010). Genetic dissection of the role of ethylene in regulating auxin-dependent lateral and adventitious root formation in tomato. Plant J..

[40] Druege U., Franken P., Lischewski S., Ahkami A.H., Zerche S., Hause B., Hajirezaei M.R. (2014). Transcriptomic analysis reveals ethylene as stimulator and auxin as regulator of adventitious root formation in petunia cuttings. Front. Plant Sci..

[41] Mergemann H., Sauter M. (2000). Ethylene induces epidermal cell death at the site of adventitious root emergence in rice. Plant Physiol..

[42] Vidoz M.L., Loreti E., Mensuali A., Alpi A., Perata P. (2010). Hormonal interplay during adventitious root formation in flooded tomato plants. Plant J..

[43] Verstraeten I., Schotte S., Geelen D. (2014). Hypocotyl adventitious root organogenesis differs from lateral root development. Front. Plant Sci..

[44] Sasidharan R., Voesenek L.A.C.J. (2015). Ethylene-mediated acclimations to flooding stress. Plant Physiol..

[45] Qi X., Li Q., Ma X., Qian C., Wang H., Ren N., Shen C., Huang S., Xu X., Xu Q. (2019). Waterlogging-induced adventitious root formation in cucumber is regulated by ethylene and auxin through reactive oxygen species signalling. Plant Cell Environ..

[46] Minami A., Yano K., Gamuyao R., Nagai K., Kuroha T., Ayano M., Nakamori M., Koike M., Kondo Y., Niimi Y. (2018). Time-course transcriptomics analysis reveals key responses of submerged deepwater rice to flooding. Plant Physiol..

[47] Xu X., Ji J., Xu Q., Qi X., Weng Y., Chen X. (2018). The major-effect quantitative trait locus CsARN6.1 encodes an AAA ATPase domain-containing protein that is associated with waterlogging stress tolerance by promoting adventitious root formation. Plant J..

[48] Prusty A., Panchal A., Singh R.K., Prasad M. (2024). Major transcription factor families at the nexus of regulating abiotic stress response in millets: A comprehensive review. Planta.

[49] Ma Z., Hu L., Jiang W. (2024). Understanding AP2/ERF transcription factor responses and tolerance to various abiotic stresses in plants: A comprehensive review. Int. J. Mol. Sci..

[50] Xu X., Chen M., Ji J., Xu Q., Qi X., Chen X. (2017). Comparative RNA-seq based transcriptome profiling of waterlogging response in cucumber hypocotyls reveals novel insights into the de novo adventitious root primordia initiation. BMC Plant Biol..

[51] Ren C.G., Kong C.C., Yan K., Zhang H., Luo Y.M., Xie Z.H. (2017). Elucidation of the molecular responses to waterlogging in *Sesbania cannabina* roots by transcriptome profiling. Sci. Rep..

[52] Zhao H., Wang Y., Zhao S., Fu Y., Zhu L. (2021). HOMEOBOX PROTEIN 24 mediates the conversion of indole-3-butyric acid to indole-3-acetic acid to promote root hair elongation. New Phytol..

[53] Xu X., Zhou H., Yang Q., Yang Y., Pu X. (2024). ZF-HD gene family in rapeseed (*Brassica napus* L.): Genome-wide identification, phylogeny, evolutionary expansion and expression analyses. BMC Genom..

[54] Varas E., Valladares S., Vielba J., Vidal N., Sanchez C. (2023). Expression of *CsSCL1* and rooting response in chestnut leaves are dependent on the auxin polar transport and the ontogenetic origin of the tissues. Plants.

[55] Wang S., Wu H., Zhang Y., Sun G., Qian W., Qu F., Zhang X., Hu J. (2024). Transcriptome reveals the regulation of exogenous auxin inducing rooting of non-rooting callus of tea cuttings. Int. J. Mol. Sci..

[56] Tian H., Fan G., Xiong X., Wang H., Zhang S., Geng G. (2024). Characterization and transformation of the *CabHLH18* gene from hot pepper to enhance waterlogging tolerance. Front. Plant Sci..

[57] Gao F., Dubos C. (2024). The arabidopsis bHLH transcription factor family. Trends Plant Sci..

[58] He N., Umer M.J., Yuan P., Wang W., Zhu H., Zhao S., Lu X., Xing Y., Gong C., Liu W. (2022). Expression dynamics of metabolites in diploid and triploid watermelon in response to flooding. PeerJ.

[59] Mosblech A., Feussner I., Heilmann I. (2009). Oxylipins: Structurally diverse metabolites from fatty acid oxidation. Plant Physiol. Bioch..

[60] Wasternack C., Feussner I. (2018). The oxylipin pathways: Biochemistry and function. Annu. Rev. Plant Biol..

[61] Shajar F., Nabi A.U., Manzoor A., Saleem S., Mushtaq N.U., Manzoor S., Rehman R.U. (2026). Understanding lipid-mediated defense mechanisms in plant responses to abiotic stress. Nucleus.

[62] Guo Z., He S., Zhong X., Yang N., Xu D. (2025). Optimizing plant alkaloid biosynthesis under drought stress: Regulatory mechanisms and biotechnological strategies. J. Plant Physiol..

[63] Kumari M., Sharma P., Singh A. (2025). Pipecolic acid: A positive regulator of systemic acquired resistance and plant immunity. BBA-Gen. Subj..

[64] Koc F.N., Dinler B.S. (2022). Pipecolic acid in plants: Biosynthesis, signalling, and role under stress. Botanica.

[65] Yang Y., Saand M.A., Huang L., Abdelaal W.B., Zhang J., Wu Y., Li J., Sirohi M.H., Wang F. (2021). Applications of multi-omics technologies for crop improvement. Front. Plant Sci..

[66] Agarwal P., Fischer H.D., Camalle M.D., Skirycz A. (2025). Not to be overlooked: Dipeptides and their role in plant stress resilience. J. Exp. Bot..

[67] Chen H., Wang Y., Song Y., Fan K., Sun L., Wang S., Yin X., Qian W., Shen J., Ding Z. (2026). Synergistic effect of small molecule oligopeptides and rhamnolipids on enhancing cold stress tolerance in tea plants: Insights from physiological and transcriptome analyses. J. Sci. Food Agric..

[68] Peretto R., Favaron F., Bettini V., De Lorenzo G., Marini S., Alghisi P., Cervone F., Bonfante P. (1992). Expression and localization of polygalacturonase during the outgrowth of lateral roots in *Allium porrum* L.. Planta.

[69] Safran J., Tabi W., Ung V., Lemaire A., Habrylo O., Bouckaert J., Rouffle M., Voxeur A., Pongrac P., Bassard S. (2023). Plant polygalacturonase structures specify enzyme dynamics and processivities to fine-tune cell wall pectins. Plant Cell.

[70] Xiao C., Somerville C., Anderson C.T. (2014). POLYGALACTURONASE INVOLVED IN EXPANSION1 functions in cell elongation and flower development in *Arabidopsis*. Plant Cell.

[71] Nagayama T., Tatsumi A., Nakamura A., Yamaji N., Satoh S., Furukawa J., Iwai H. (2022). Effects of polygalacturonase overexpression on pectin distribution in the elongation zones of roots under aluminium stress. AoB Plants.

