# Supplementary material for: Integrated Metabolomic and Proteomic Analyses of Adventitious Rooting in Cucumis melo Under Waterlogging Stress
Source: Biology (Basel). 2026 Jul 17;15(14):1185. doi: 10.3390/biology15141185 (PMC13405407; doi:10.3390/biology15141185)
Supplement: Supplementary file 1 [file biology-15-01185-s001.zip › Supplementary Figures.pdf]

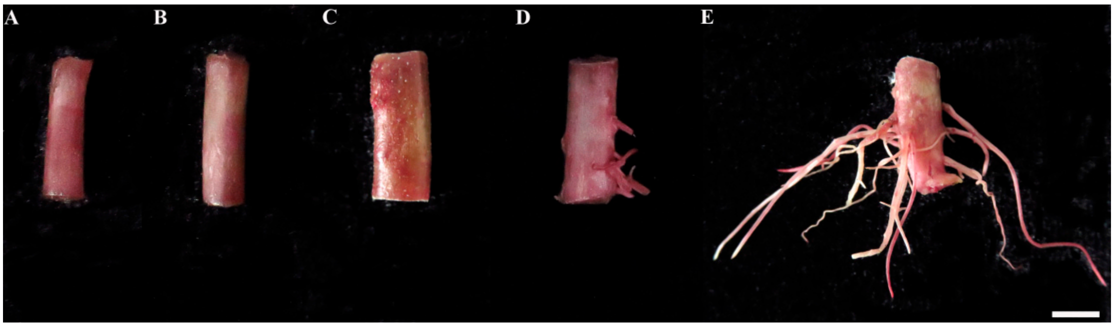

Figure S1. Morphological observation of adventitious root development in melon under waterlogging stress. **(A)** Photograph of hypocotyl at 0 h after waterlogging (HAW); **(B)** 24 HAW; **(C)** 48 HAW; **(D)** 72 HAW; **(E)** 96 HAW. Bars, 5 mm.

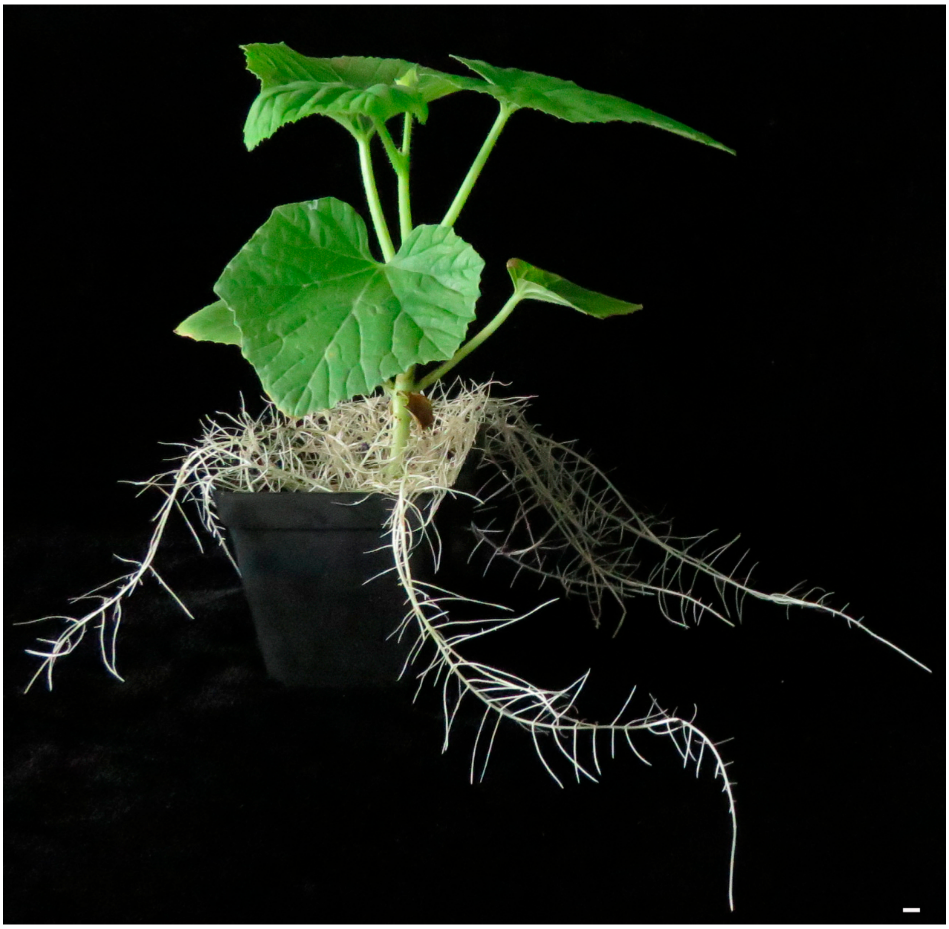

Figure S2. Morphological phenotypes of melon seedlings photographed at 7 days after waterlogging stress. Bars, 1 cm.

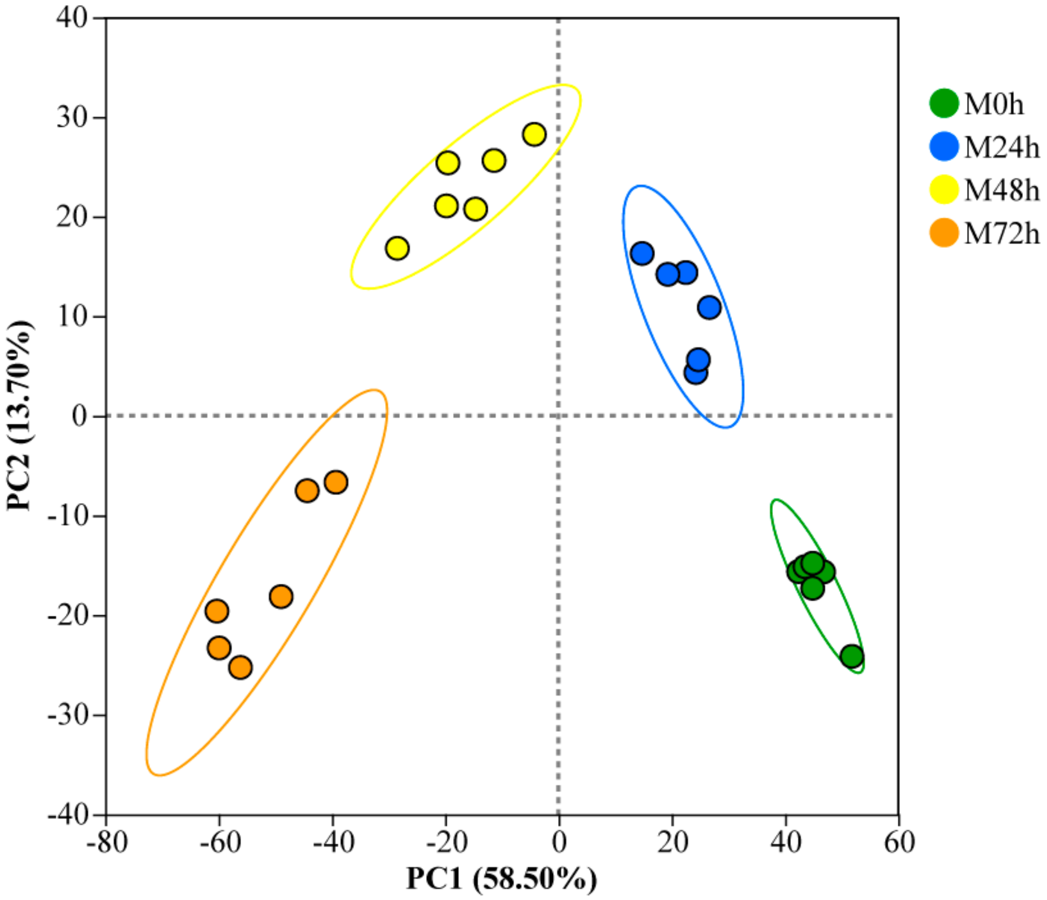

Figure S3. Principal component analysis (PCA) plots of the untargeted metabolomic data.

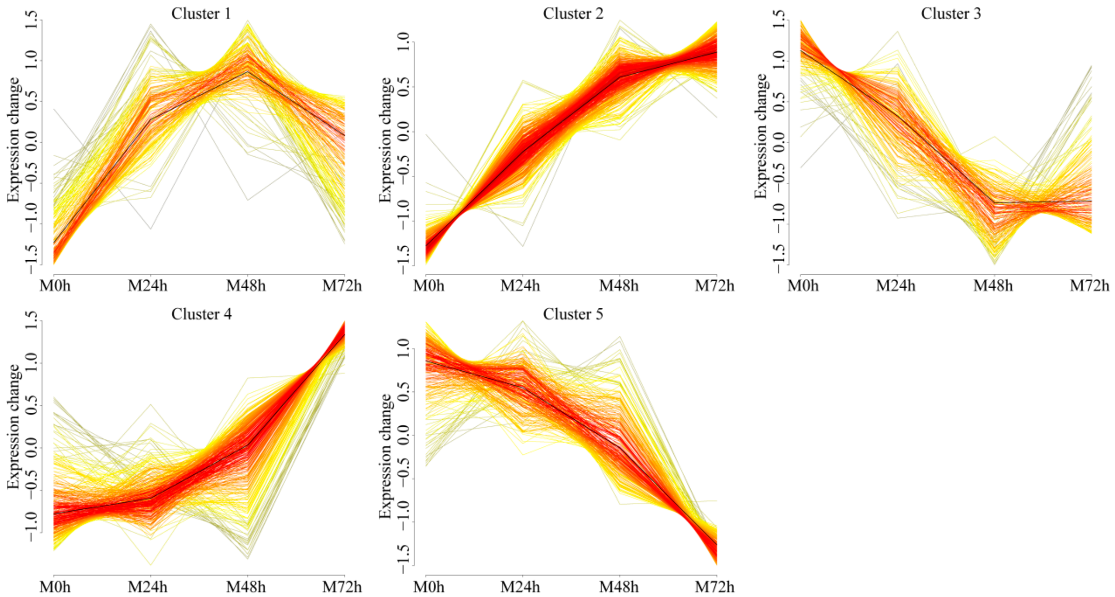

Figure S4. A cluster analysis of all differentially accumulated metabolites (DAMs).

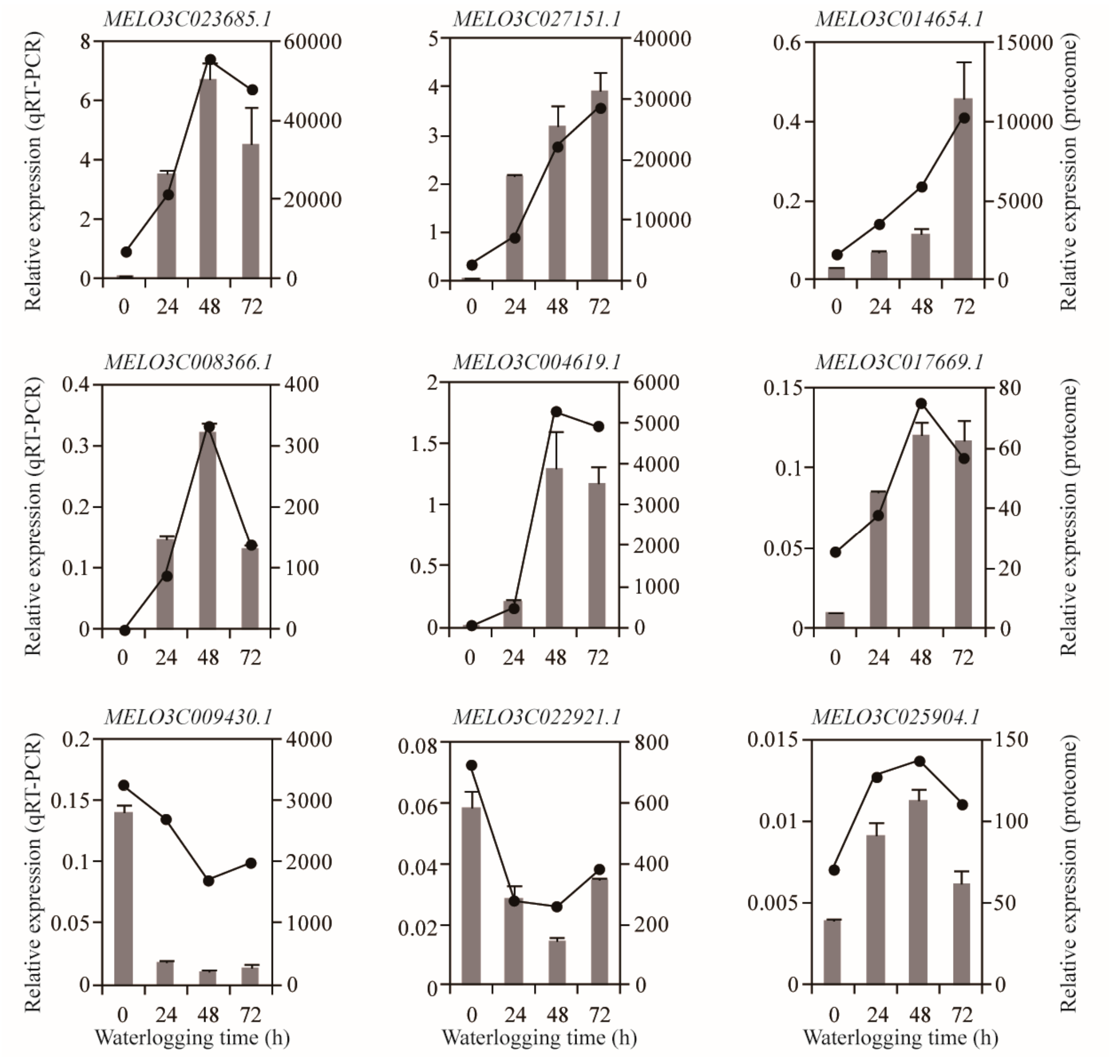

Figure S5. Quantitative reverse transcription-PCR (qRT-PCR) verification of some key genes in proteome. The histograms denote qRT-PCR results and line graphs represent proteomic data.
